# Initial Changes in Clinical Practice Following New Guidlines on the Use of Valproate Products – a Study in Older Adult Inpatients

**DOI:** 10.1192/bjo.2025.10173

**Published:** 2025-06-20

**Authors:** Mohammad Faizan Lodhi

**Affiliations:** North Staffordshire Combined Healthcare NHS Trust, Stoke on Trent, United Kingdom

## Abstract

**Aims:** To assess the impact of newly implemented guidelines on the prescribing practices of valproate products in a clinical setting. Specifically, comparing the proportions of inpatients over 65 on valproate before and after the new guidelines were implemented in January 2024.

**Methods:** This was a retrospective comparative study involving 2 patient cohorts admitted on the older adults ward at the North Staffordshire Combined Healthcare NHS Trust in Stoke-On-Trent, Staffordshire. **Cohort 1**: 30 patients who were admitted from October to December 2023 (approximately 1 month prior to implementation of new guidelines). **Cohort 2:** 30 patients who were admitted from July to September 2024 (approximately 6 months after implementation of new guidelines). Statistical significance between the 2 groups was determined using the Chi-square test.

**Results:** A notable decrease (16.7% to 6.7%) was observed in the percentage of patients who were admitted on valproate after the new guidelines were implemented (2 out of 30) as compared with the group admitted prior to their implementation (5 out of 30). However, based on a Chi- square test, the proportion of patients prescribed sodium valproate before and after the implementation of new guidelines is not statistically significant in this study. This reduction may suggest that, although, not yet statistically significant, the new guidelines are influencing prescribing practices regarding valproate products.

**Conclusion:** Although not statistically significant in this study, these findings suggest that there may be a trend toward reduced prescribing of valproate products after the introduction of these new treatment guidelines. This trend could be attributed to increased awareness among healthcare providers regarding the risks associated with valproate products. However, this study was limited by its sample size, scope and age group of participants, therefore, further research is warranted to explore more extensive datasets and longer follow-up periods. Such studies would provide a clearer understanding of the long-term influence of implementing these new guidelines on prescribing practices and patient safety.

